# Low-Level Visual Information Is Maintained across Saccades, Allowing for a Postsaccadic Handoff between Visual Areas

**DOI:** 10.1523/JNEUROSCI.1169-20.2020

**Published:** 2020-12-02

**Authors:** Jasper H. Fabius, Alessio Fracasso, David J. Acunzo, Stefan Van der Stigchel, David Melcher

**Affiliations:** ^1^Institute of Neuroscience and Psychology, College of Medical, Veterinary and Life Sciences, University of Glasgow, Glasgow G12 8QQ, United Kingdom; ^2^Center for Mind/Brain Sciences and Department of Psychology and Cognitive Sciences, University of Trento, I-38122 Trento, Italy; ^3^Experimental Psychology, Helmholtz Institute, Utrecht University, 3584 CS, Utrecht, The Netherlands; ^4^Psychology Program, Division of Science, New York University Abu Dhabi, Abu Dhabi, United Arab Emirates

**Keywords:** magnetoencephalography, multivariate pattern analysis, saccades, vision, visual stability

## Abstract

Experience seems continuous and detailed despite saccadic eye movements changing retinal input several times per second. There is debate whether neural signals related to updating across saccades contain information about stimulus features, or only location pointers without visual details. We investigated the time course of low-level visual information processing across saccades by decoding the spatial frequency of a stationary stimulus that changed from one visual hemifield to the other because of a horizontal saccadic eye movement. We recorded magnetoencephalography while human subjects (both sexes) monitored the orientation of a grating stimulus, making spatial frequency task irrelevant. Separate trials, in which subjects maintained fixation, were used to train a classifier, whose performance was then tested on saccade trials. Decoding performance showed that spatial frequency information of the presaccadic stimulus remained present for ∼200 ms after the saccade, transcending retinotopic specificity. Postsaccadic information ramped up rapidly after saccade offset. There was an overlap of over 100 ms during which decoding was significant from both presaccadic and postsaccadic processing areas. This suggests that the apparent richness of perception across saccades may be supported by the continuous availability of low-level information with a “soft handoff” of information during the initial processing sweep of the new fixation.

**SIGNIFICANCE STATEMENT** Saccades create frequent discontinuities in visual input, yet perception appears stable and continuous. How is this discontinuous input processed resulting in visual stability? Previous studies have focused on presaccadic remapping. Here we examined the time course of processing of low-level visual information (spatial frequency) across saccades with magnetoencephalography. The results suggest that spatial frequency information is not predictively remapped but also is not discarded. Instead, they suggest a soft handoff over time between different visual areas, making this information continuously available across the saccade. Information about the presaccadic stimulus remains available, while the information about the postsaccadic stimulus has also become available. The simultaneous availability of both the presaccadic and postsaccadic information could enable rich and continuous perception across saccades.

## Introduction

How the world appears stable despite making several saccades every second, dramatically changing the retinal image, remains a mystery in neuroscience. This introspective stability is correlated with psychophysical data, where responses to a postsaccadic stimulus are affected by a presaccadic stimulus when presented at the same spatiotopic location, but—because of the saccade—at a different retinotopic location (Prime et al., 2007; Wittenberg et al., 2008; Demeyer et al., 2009; Fracasso et al., 2010; Ganmor et al., 2015; Oostwoud Wijdenes et al., 2015; Wolf and Schütz, 2015; Eymond et al., 2019; Fabius et al., 2019). Given that the visual system is retinotopically organized (Wandell et al., 2007), this raises the question of how perceptual continuity is established.

Previous studies examined (neural) responses to briefly flashed stimuli, and formulated accounts of visual stability in the form of remapping of receptive fields or remapping of attentional pointers (Duhamel et al., 1992; Cavanagh et al., 2010; Mirpour and Bisley, 2016). Conceptually, these imply that the spatial receptive field profile of neurons is altered around the time of a saccade to counteract the change in retinal input caused by a saccade. Alternatively, the stability of attentional pointers has been explained by a handoff in attentional gain modulations of neural responses: the dual-spotlight theory of attentional updating (Marino and Mazer, 2018; Golomb, 2019).

Under both the account of remapping of receptive fields and the dual-spotlight theory, it remains debated whether and how visual feature information is maintained across saccades. Although it has been argued that visual feature information must be partially maintained, while irrelevant features will be discarded (Pollatsek et al., 1984; Irwin, 1992; Melcher and Colby, 2008; Prime et al., 2011; Cha and Chong, 2014), it is currently unclear whether low-level information is indeed maintained across saccades and what could be the underlying neural mechanism.

In many previous studies on the updating of visual features, differences in behavior or neural responses were examined using a stimulus that suddenly appears at an attended versus unattended location or that changes versus remains constant across saccades, violating visual stability. However, there is both behavioral and neurophysiological evidence suggesting that the degree of visual stability is context dependent (McConkie and Currie, 1996; Churan et al., 2011; Lisi et al., 2015; Atsma et al., 2016; Rao et al., 2016). Here, we examined the time course of updating of feature information that remained constant across the saccade. Specifically, we investigated spatial frequency (SF) processing across saccades. SF information is thought to be important for the perception of scene gist (Henderson and Hollingworth, 1999; Oliva and Torralba, 2001) and object identity (Bar et al., 2006), and is linked to subjective experience of a richly detailed scene (Sahraie et al., 2003, 2010; Cohen et al., 2016; Burra et al., 2019).

**Figure 1.**
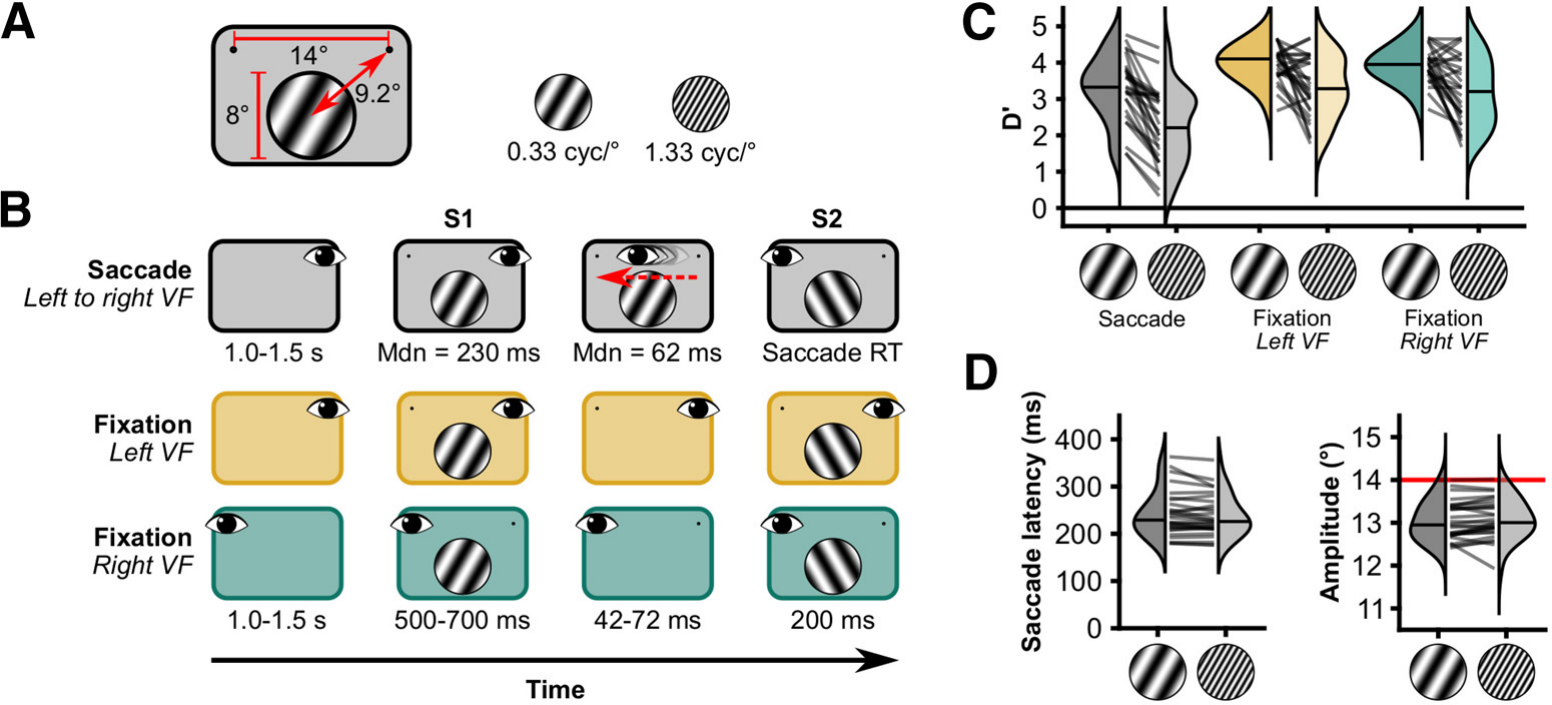
Stimuli, experimental paradigm and behavioral results. ***A***, Stimuli were sinusoidal gratings (diameter, 8°) presented 9.2° from a fixation point to the upper left or right of the grating. The spatial frequency was either 0.33 cycle/° or 1.33 cycles/°. ***B***, Trial timeline. Subjects completed three conditions of an orientation change detection task. In each condition, the task was to indicate whether the orientation changed from S1 to S2. The change in orientation was always 60° and occurred on half of the trials. In Saccade trials, saccades were always made from right to left, and the orientation could be changed during the saccade. In Fixation trials, a blank period of 42–72 ms was presented between S1 and S2. Saccade and Fixation trials were presented in separate blocks. Left and right fixation trials were randomly interleaved. ***C***, Change detection performance. Lines represent individual subjects. Horizontal lines in distribution patches represent median D′. ***D***, Oculomotor performance. cyc, Cycles; Mdn, median.

Human subjects made saccades across a grating with a constant low or high SF (Fig. 1*A*) while we recorded eye position and magnetoencephalography (MEG). We quantified SF information from the MEG data using classifiers that were trained on data from two fixation conditions (Fig. 1*B*). The results suggest presaccadic SF information remains available after saccade offset, while postsaccadic SF information builds quickly, prompting the hypothesis that higher brain areas could read out the SF of the stimulus during the entire interval. The synchronous presence of both presaccadic and postsaccadic stimulus SF could enable rich and continuous visual perception across saccades.

## Materials and Methods

We analyzed data from 29 human subjects (13 females; mean age, 25.3 years; age range, 20–35 years; 23 subjects were right handed). All subjects had normal or corrected-to-normal vision. We collected data from two more subjects, but their data could not be included in the analysis because of technical issues (*n* = 1) and an inability to perform the task (*n* = 1). In the latter case, the subject was making saccades to the stimulus rather than the saccade target. Of the 29 subjects, one subject was excluded after analyzing the behavioral performance. Performance of this subject in the saccade condition on the orientation change detection task was at chance level both for 0.33 cycle/° (D′ = 0.05) and 1.33 cycles/° (D′ = 0.11) stimuli. Informed consent was given by all participants. Experimental procedures were reviewed and approved by the local ethics committee of the University of Trento. All experiment scripts, data, and analysis scripts are available on Open Science Framework (https://doi.org/10.17605/OSF.IO/NGUD8).

### 

#### Setup

Subjects were dressed in scrubs. Five head position indicator coils were attached. Head coordinate frame, coil position, and head shape were determined with the FASTRACK 3D digitization system (Polhemus) using the left and right preauricular points, the nasion, and 500 points distributed across the head. Head position was measured at the beginning of each experimental run. MEG data were acquired with a Vectorview 306 channel MEG machine (Neuromag, Elekta). Eye position data were acquired with an Eyelink 1000+ at 1000 Hz, recording the left eye (SR Research). Stimuli were projected with a PROPixx projector (VPixx Technologies) onto a translucent screen 100 cm away from the subject, with a refresh rate of 120 Hz. The display size was 51 by 38 cm, with a resolution of 1440 by 1080 pixels. Visual onsets were monitored with a photodiode, placed in the lower left corner of the display over a small square that changed polarity with every change in display. Manual responses were recorded with RESPONSEPixx (VPixx Technologies). Four electrooculography (EOG) and two electrocardiography (EKG) electrodes were attached, but these recordings were not used in the analysis. Electrodes were placed above and below the left eye for measuring the vertical EOG, and on the outer canthi for measuring the horizontal EOG. EKG was recorded using Einthoven's lead II, which used the left leg and the right arm electrodes.

#### Stimuli

Stimulus presentation was controlled with MATLAB Psychtoolbox 3 (Brainard, 1997; Pelli, 1997; Kleiner et al., 2007) and its DATAPixx extension (VPixx Technologies). The Eyelink extension of the Psychtoolbox (Cornelissen et al., 2002) was used to control the eye tracker and control the gaze-contingent display. The stimuli were static sinusoidal gratings (diameter, 8° visual angle; orientation, −30° or 30° from vertical; spatial frequency, 0.33 or 1.33 cycles/°; phase, 0 or π, to keep luminance equal). Stimuli were presented at full contrast (black, 1.94 cd/m^2^; white, 142 cd/m^2^) on a uniform gray background (61.1 cd/m^2^). Stimulus contrast was reduced to zero over the outer 0.6° with a raised cosine envelope. The center of the stimuli was located 6° below the horizontal meridian and was horizontally centered on the display. The fixation points consisted of black dots (radius, 0.5°) overlaid with a gray cross and a black point (radius, 0.07°) in the center of the cross (Thaler et al., 2013). Fixation points were located 7° to the left or right from the center of the display.

#### Procedures

##### Saccade condition: Sac-left visual field

From a saccade perspective, subjects performed trials in two different conditions, a Saccade and a Fixation condition (Fig. 1*B*). In the Saccade condition, subjects performed a trans-saccadic change detection task on the orientation of the stimulus. In these trials (416 trials/subject), subjects initially fixated the right fixation point for a random duration of 1.0–1.5 s (uniformly distributed). Then stimulus 1 (S1) appeared in the left visual field (LVF), together with the second fixation point. Subject made a saccade (required amplitude, 14°) to the left fixation point immediately after stimulus onset. In a pilot dataset, we observed that this procedure gave rise to median saccade latencies of ∼0.2 s. The maximum (max) saccade latency during the experiment was 1.0 s. If subjects had not executed a saccade by then, text was displayed encouraging them to make faster saccades. During the saccade, stimulus 2 (S2) was presented. S2 onset was determined gaze contingently during the experiment (i.e., when the gaze was >2° from the fixation point). S2 had either the same orientation as S1 or a 60° different orientation. That is, if S1 had an orientation of −30° from vertical and the orientation changed during the saccade, S2 would have an orientation of +30°. We only used these two orientations. S2 was presented for the same duration as S1. After the saccade, subjects manually indicated whether S1 and S2 had the same orientation. The maximum response latency during the experiment was 2.0 s. If subjects had not responded by then, a text was displayed encouraging them to make faster responses. We abbreviate this condition as Sac-LVF, because this condition consists of trials where subjects made saccades and S1 appeared in the left visual field.

##### Saccade condition: Sac-no VF

Additionally, we included trials without a stimulus (208 trials/subject). In these trials, subjects fixated the right fixation point for 1.0–1.5 s before the left fixation point appeared. Subjects made a saccade to the left fixation point. When a saccade was detected, the trial ended after a time equal to the sum of the online saccade latency and an additional 0.5 s. Subjects did not give a manual response in these trials. These “saccade, no stimulus” trials were mixed with the trans-saccadic change detection trials. Online saccade detection was position based (i.e., a “saccade” was detected as soon as gaze was outside an area of 2° around the right fixation point). For the analysis, saccades were detected offline using a velocity-based algorithm (see MEG analysis, Preprocessing).

##### Fixation conditions: Fix-LVF/Fix-right visual field

In the Fixation condition, the subject also performed a change detection task, similar to the trans-saccadic change detection task (416 trials/subject). Subjects fixated the left or right fixation point for the entire length of a single trial. S1 was presented for a random duration between 0.5 and 0.7 s (uniformly distributed) in the center of the screen. Then, S1 was removed for a duration between 42 and 75 ms (normally distributed; mean, 55 ms; SD, 6; quantized by the 120 Hz refresh rate of the projector) and followed by S2 presented with the same duration as S1. The duration between S1 and S2 was matched to the duration of saccades from the pilot data. To stay close to the visual processing we aim to study, we refer to the condition where the stimulus appeared in the right visual field (RVF; i.e., confusingly, when subjects were fixation the left side of the screen) as Fix-RVF, and the other fixation condition as Fix-LVF because there the stimulus appeared in the left visual field. Note that in the Saccade condition, the stimulus first appeared in the left visual field (hence, we use the abbreviation Sac-LVF), but was brought into the right visual field as a consequence of the saccade.

##### Block design

The Saccade and Fixation conditions were presented in separate blocks. Subjects performed 13 blocks of the Saccade condition, and 13 blocks of the Fixation condition. The order of conditions (i.e., fixation first or saccade first) was balanced between subjects. The parameters spatial frequency (high/low), base orientation (−30°/30°), grating phase (0/π), and change presence (with/without) were factorially presented within each block. In the Saccade condition, trials without a stimulus were implemented as a spatial frequency of 0 in this factorization. In the Saccade condition, all factorial combinations were repeated twice within a block, resulting in 48 trials per block. In the Fixation condition, fixation location (left/right) was included as an additional parameter in the factorization, resulting in 32 trials per block. One block of the Saccade condition and one block of the Fixation condition were combined into one experimental run. The duration of one run was ∼8 min. Before the experiment started, subjects performed one block of the Fixation condition and one block of the Saccade condition as practice. The Fixation condition was always practiced first.

#### Behavioral analysis

##### Change detection

We assessed orientation change detection performance by computing D′ (“D-prime”) per subject and condition.

##### Eye-tracking data processing

The raw Eyelink recordings in the MEG datafile were converted from volts to pixels. We observed a small but consistent lag between the recordings in the MEG datafile and the Eyelink datafile of 7 ms. This lag probably originated during the digital-to-analog conversion and was compensated for by shifting all Eyelink data in the MEG datafile with 7 ms back in time with respect to the MEG data. Saccades were detected with the saccade detection algorithm of Nyström and Holmqvist (2010), with a minimum (min) fixation duration of 40 ms and a minimum saccade duration of 10 ms. To determine the onset of a visual event, we converted the raw photodiode signal to a ternary signal—because we used three gray values: black, gray and white—by taking four linearly separated values between the minimum and maximum values of the raw signal. All values below the second boundaries were classified as black (−1). All values between the second and third boundaries were classified as gray (0). All values higher than the third boundary were classified as white (1). The absolute difference of the trinary signal was used to obtain the timing of a visual onset. We computed the median latency and amplitude per subject and per condition.

#### MEG analysis

##### Preprocessing

We visually inspected all data and marked noisy channels. The native Maxwell filter of the Neuromag (Elekta) was applied to filter signals that originated outside the MEG helmet (Taulu et al., 2004, 2005; Taulu and Kajola, 2005). Line noise (50 Hz) and its harmonics (100 and 150 Hz) were attenuated using a Discrete fourier transform filter on the continuous data of each run. Data were then cut into epochs from 0.5 s before until 1.5 s after S1 onset. Then, data were downsampled to 500 Hz for the event-related fields (ERFs) and to 250 Hz for the multivariate analyses. We applied as little preprocessing as possible to minimize the risk of introducing systematic differences in the data that could be exploited in the multivariate analyses.

##### Epoch exclusion

All epochs from −0.5 to 1.5 s after S1 onset were visually inspected for remaining MEG artifacts (e.g., muscle activity). Epochs containing artifacts were removed (mean, 3.9%; min, 0.4%; max, 7.3% of epochs per subject). In the conditions with saccades, epochs were included only if (1) there was a single saccade after S1 onset and before S2 onset; (2) the saccade end point was at least 4° over the vertical midline of the screen, bringing the stimulus from being entirely in the left visual field to entirely in the right visual field; and (3) the saccade end point was higher than 2° below the horizontal midline of the screen, keeping the stimulus entirely in the lower visual field (mean, 8.2%; min, 0.2%; max, 28.8% rejected). In the Fixation conditions, epochs were included only if subjects (1) maintained gaze within an area of 2° visual angle around the fixation point during the entire epoch and (2) did not make microsaccades with amplitudes >0.5° (mean, 4.2%; min, 0.1%; max, 21.4% rejected). After defining valid epochs, we further included epochs in the saccade condition only when the saccade latency was between 150 and 500 ms. These latency values were selected because we intended to compare the saccade and fixation conditions. The duration of S1 in the fixation conditions was minimally 500 ms. The lower bound of the latency inclusion was motivated both by theoretical reasons, since we wanted to only include trials in which there was sufficient time to visually process S1 for the change detection task, and by the desire to have epochs of a considerable length for the data analysis.

##### Event-related planar gradients

**Figure 2.**
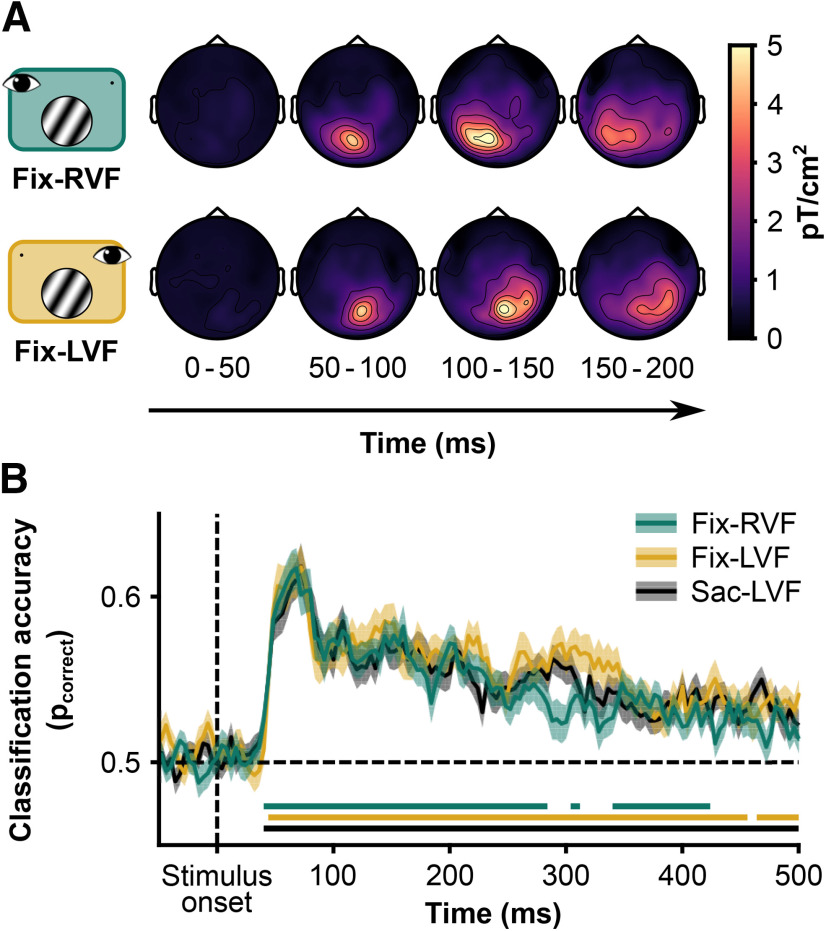
***A***, Evoked planar fields locked to stimulus onset. When the grating was presented in the right VF (top row), the evoked field was centered over the left posterior sensors. In contrast, when the grating was presented in the left VF (bottom row), the evoked field was centered over the right posterior sensors (i.e., in both cases), the grating evoked a contralateral response, starting ∼40 ms after stimulus onset. ***B***, Cross-validated classification performance (10-fold) per fixation condition of spatial frequency. Shading is 1 SEM across subjects. Horizontal lines indicate significant deviations from 0.5 (two-sided, α = 0.05, corrected for multiple comparisons using permutation tests (*n* = 1 × 10^4^) and threshold-free cluster enhancement). Fix-RVF: peak accuracy = 0.62, at 68 ms after stimulus onset; Fix-LVF: peak accuracy = 0.62, at 72 ms after stimulus onset; Sac-LVF: peak accuracy = 0.62, at 72 after stimulus onset.

Event-related planar gradients were computed using the combined gradiometer data with the recordings locked to saccade onset. We used planar gradiometers because their measurements allow for a direct distinction between left and right hemisphere activity, whereas magnetometers do not. We used data locked to stimulus onset, with baseline normalization over a baseline from −0.2 to 0 before stimulus onset. In addition, we locked data from the Saccade conditions to saccade offset with a window from −0.6 to 0.4 after saccade offset. For this alignment, the data were normalized to the window of −0.1 to −0.004 before saccade offset. Then we computed the average per sensor and subsequently combined the averaged gradiometers. Last, we subtracted the average activity in the baseline period. We did not apply any filters before or after computing the planar gradients other than described in Preprocessing. Topographic maps of ERFs are displayed in Figure 2*A*.

Univariate differences between stimuli with a high or low SF were computed for both planar gradiometers and magnetometers. Again, we computed the average per sensor, then combined planar gradiometers, performed a baseline correction (subtracting the average between −0.2 and −0.004 ms before stimulus onset), and finally subtracted the responses evoked by low SF stimuli from the responses evoked by high stimuli.

Because behavioral performance was high, we also examined the difference between evoked fields after saccade offset for trials when the orientation stayed the same and trials where the orientation changed during the saccade. For each subject, we computed planar gradients in a manner similar to the procedure described above, but we aligned the data to saccade offset and used a baseline period from −0.1 to −0.004 before saccade offset (i.e., in the saccade window). We then subtracted the gradients from trials without a change from the gradients with a change and tested the difference against 0.

##### Multivariate pattern analysis

We performed two different types multivariate pattern analyses (MVPAs). All MVPAs were performed using the CoSMoMVPA toolbox for MATLAB (Oosterhof et al., 2016). We used all 306 channels to train linear support vector machines (SVMs), similar to previous studies (Ramkumar et al., 2013; Cichy et al., 2015). Each MVPA was performed on the level of single subjects. First, we assessed whether any stimulus features could be decoded from each condition separately. Second, we examined to which extent classifiers trained on one condition could decode spatial frequency in another condition. We performed cross-condition classification of spatial frequency. Here, SVMs were trained on the MEG data of either the Fix-LVF or the Fix-RVF condition to test for spatial invariance of the classification of spatial frequency. Subsequently, we tested on data of the Sac-LVF and Fix-RVF, or Sac-LVF and Fix-LVF conditions, respectively. Because trials in the training and test set were independent, we did not use cross-validation here. The same preprocessing and temporal searchlight parameters as in the first MVPA analysis were used.

##### Within-condition spatial frequency classification

We performed 10-fold cross-validation of linear SVMs trained to separate stimulus features (spatial frequency, orientation, and phase) from the data of the Fixation conditions (Fig. 2*B*). For this analysis, the MEG data were aligned to the onset S1, processed at 250 Hz, and standardized to a baseline period from −0.2 to 0 s before S1 onset. We used a “temporal searchlight” with a radius of 8 ms (i.e., two samples at 250 Hz). This temporal searchlight means that for each time point, classification is based not only on the data at that time point but also on 10 neighboring timepoints. In each fold of the cross-validation, trials were balanced for the stimulus feature that would be classified.

##### Cross-condition temporal generalization of spatial frequency classification

We examined the temporal generalization of cross-condition classification of spatial frequency. In other words, we tested to what extent classification based on training with one condition (Fix-RVF or Fix-LVF) transferred to the testing set (Sac-LVF) for different points in time. The SVMs were trained on data from the Fixation conditions and tested on the data from the Saccade condition. Data were baseline standardized to −0.2 and 0 s before S1 onset. We used a temporal searchlight with a radius of 8 ms (i.e., two samples). The test data from the Sac-LVF condition were aligned to S1 onset for one temporal generalization matrix and aligned to saccade offset for the second. Thus, in total four temporal generalizations were made per subject. With this analysis, we examined how stimulus-related activity changes across a saccade, and whether this progression resembles activity related to stimulus onsets under stable fixation at the presaccadic or the postsaccadic fixation location.

##### Time course of presaccadic and postsaccadic spatial frequency representation

We extracted diagonal bands from the temporal generalization matrices (see Fig. 5*A*). These diagonal bands had their origin either at stimulus onset in both the Sac-LVF and Fixation conditions or at saccade offset in the Sac-LVF condition and stimulus onset in the Fixation conditions. The two diagonals represent the similarity between stimulus-evoked responses after stimulus onset and after saccade offset. Additionally, we computed a third diagonal using the matrix where the Sac-LVF condition was locked to saccade offset. This diagonal had its origin at saccade offset in the Sac-LVF and at stimulus onset plus the median saccade latency plus the median saccade duration. This shift was computed for each subject separately to account for variations in median saccade latencies and durations. The width of the diagonal bands was 20 ms or five samples (at 250 Hz).

##### Spatial specificity of spatial frequency information

To assess the spatial specificity of the cross-condition classification, we compared how accurately the Fix-LVF classifier was able to decode SF from the Fix-RVF condition (i.e., the other fixation condition) compared with the Sac-LVF condition. For this analysis, the above-trained classifiers were used to decode spatial frequency from the other fixation condition (i.e., using the Fix-RVF classifier to decode the Fix-LVF condition, and conversely).

##### Presaccadic updating of spatial frequency information

We examined the hypothesis of presaccadic transfer of spatial frequency information. To this end, we used the same classifiers from the cross-condition temporal generalization (i.e., trained on the Fix-LVF and Fix-RVF data) and used them to classify spatial frequency from the Saccade condition with trials aligned to saccade onset. If the classifier trained on the Fix-RVF data would be able to classify spatial frequency above the chance level, this would be indicative of presaccadic updating.

##### Diagonal width specificity of cross-condition temporal generalization

To examine the specificity of our results to the width of the diagonal band in the results of the cross-condition temporal generalization, we determined whether the patterns for diagonal bands with different widths were similar. In general, a representation that develops rapidly across time will yield limited temporal generalization of classification (King and Dehaene, 2014). Thus, the width of the diagonal extracted from the temporal generalization matrix will strongly affect the average of the diagonal. In contrast, a representation that remains stable across time will yield high temporal generalization. In that case, the width of the diagonal will not affect the average of the diagonal.

##### Bias in cross-condition temporal generalization

To assess a potential bias in spatial frequency cross-condition classification that could be introduced by the execution of a saccade, we performed another cross-condition temporal generalization analysis. For this analysis, we used the classifiers trained on the two Fixation conditions and assessed their performance on trials from the Sac-no VF condition. The test data were aligned to saccade offset. Instead of analyzing classification performance as the proportion of correct classifications, we analyzed the proportion of trials on which the high spatial frequency was chosen. We used the same statistics to analyze classification performance as we did for the other temporal generalizations.

#### Statistics

Behavioral parameters were analyzed with Bayesian repeated-measures ANOVAs with default prior settings in JASP (JASP Team, 2020). Bayes factors were computed for the fixed effects across matched models (Rouder et al., 2012). Orientation change detection performance was analyzed with a 3 × 2 design. We used the factors condition (three levels: Fix-RVF, Fix-LVF, Sac-LVF) and spatial frequency (two levels: 0.33 cycle/° and 1.33 cycles/°). The saccade parameters “latency” and “amplitude” were analyzed with a 3 × 1 design, with the factor stimulus (0.33 cycle/°, 1.33 cycles/° and none).

For the analysis of evoked planar fields, we tested for significant deviations from 0 per sensor and per time point using one-sample *t* tests (α = 0.05, two-tailed). We corrected for multiple comparisons using cluster-based permutations with threshold-free cluster enhancement (Maris and Oostenveld, 2007; Smith and Nichols, 2009). We used 1 × 10^4^ permutations, and sensors were clustered based on Delaunay triangulation. Statistics were computed with the CoSMoMVPA toolbox (Oosterhof et al., 2016).

For the MVPAs, classifier performance was assessed against chance level using one-sample *t* tests (α = 0.05, two-tailed), corrected for multiple comparisons using cluster-based permutations with threshold-free cluster enhancement. Classifier accuracy was computed as “proportion correct” but was converted to the log-odds of a correct response before entering the statistical analysis. Chance level was log-odds = 0. We used 1 × 10^4^ permutations. Time points were regarded as clusters within a radius of 8 ms (i.e., two samples at 250 Hz). Statistics were computed with the CoSMoMVPA toolbox. We also tested classifier performance for change (in orientation across the saccade) and no-change trials separately. However, since the overall pattern of results was similar, analyses are reported for all trials (change and no-change).

## Results

### Behavior

#### Orientation change detection

Overall, sensitivity for changes in orientation was high (average ± SEM D′ = 3.32 ± 0.11), but there were differences in sensitivity between the different conditions and spatial frequencies (Fig. 1*C*). There was strong evidence in favor of an effect of both condition [Bayes factor alternative over null hypothesis (BF_10_) = 1.43 × 10^10^] and spatial frequency (BF_10_ = 1.35 × 10^12^), but not for their interaction (BF_10_ = 0.402). *Post hoc* tests showed that the effect of condition was primarily driven by differences between the Sac-LVF and two Fixation conditions (BF_10_ = 4.17 × 10^6^ and BF_10_ = 7.50 × 10^7^), but not between the two Fixation conditions (BF_10_ = 0.316). Together, these results show that subjects were attending the stimulus in all conditions, but performance was better in the fixation conditions and for stimuli with a low spatial frequency.

#### Saccades

We analyzed the median saccade latencies and mean horizontal component of the saccade amplitude (Fig. 1*D*). The average ± SEM of median saccade latencies for Saccade at 0.33 cycle/° trials was 242 ± 9 ms. For Saccade at 1.33 cycles/° trials, the average median latency was 238 ± 8. These medians were computed after excluding trials with latencies <0.15 or >0.50 s. The data were inconclusive about a difference in saccade latencies between the Saccade at 0.33 cycle/° and the Saccade at 1.33 cycles/° (BF_10_ = 1.11). On average, saccades were hypometric. The average ± SEM horizontal component of amplitude in the Saccade at 0.33 cycle/° trials was 13.05 ± 0.08°, and in Saccade at 1.33 cycles/° trials, 13.09 ± 0.09°. The evidence was inconclusive about a difference between Saccade 0.33 cycle/° and Saccade 1.33 cycles/° conditions (BF_10_ = 0.532). Together, these results show that oculomotor behavior among the different Saccade conditions was similar. Subjects followed the instructions regarding the timing and magnitude of the saccadic eye movements

### Event-related planar gradients

After preprocessing the MEG data and excluding trials containing artifacts or incorrect saccades, we first examined the evoked response to S1 for the Fixation conditions (Fig. 2*A*). As expected, the presentation of the stimulus evoked an early response from occipital sensors, spreading over time into parietal and temporal sensors. Comparing the Fix-RVF and Fix-LVF conditions, the evoked responses were contralateral (Fig. 2*A*).

We examined differences in evoked planar gradients and fields between high- and low-SF stimuli. In all conditions, there was a difference early after stimulus onset in planar gradients over occipital gradiometers (Fix-RVF, 52–74 ms; Fix-LVF, 52–70 ms; Sac-LVF, 52–74 ms) and magnetometers (Fix-RVF, 50–72 ms; Sac-LVF, 52–74 ms). The early difference in evoked fields between high and low SF did not pass our significance threshold in the Fix-LVF condition. Later, there was a difference in evoked fields over central magnetometers in the Fix-LVF condition (120–174 ms) and Sac-LVF condition (142–184 ms), but not in the Fix-RVF condition. In the Sac-LVF condition, there was a brief period (158–160 ms) with a difference between evoked gradients over right temporal planar gradiometers.

Although not the primary focus of our analysis, we also examined differences in planar gradients between trials with a change in orientation and those without. Between 100 and 300ms after saccade offset, there were stronger evoked responses in parieto-occipital sensors for trials with a change than for trials without a change. In all MVPAs, we balanced the number of trials with and without changes per spatial frequency.

### Multivariate pattern analysis

#### Within-condition spatial frequency classification

We trained linear SVMs to classify the spatial frequency of the stimulus based on all MEG data (both magnetometers and gradiometers) in each condition separately. Classification performance was assessed with 10-fold cross-validation. It has previously been demonstrated that spatial frequency can be reliably decoded from MEG data, albeit in a study using larger stimuli presented around the point of fixation (Ramkumar et al., 2013). In all conditions, classification accuracy sharply increased ∼40 ms after stimulus onset, as previously observed (Ramkumar et al., 2013). We found clusters with significant above-chance classification accuracy (Fig. 2*B*), starting after 40 ms in the Fix-RVF condition, after 44 ms in the Fix-LVF condition, and after 40 ms in the Sac-LVF condition. The peak accuracy of the group average was *p*_correct_ of 0.62 in all conditions. This peak was observed after 68 ms in the Fix-RVF condition, after 72 ms in the Fix-LVF condition, and after 72 ms in the Sac-LVF condition. We repeated the same procedure in attempt to decode the orientation (−30° or 30°) and phase of the stimulus (0, π). However, classification did not rise above chance level in any of the three conditions.

#### Cross-condition temporal generalization of spatial frequency classification

**Figure 3.**
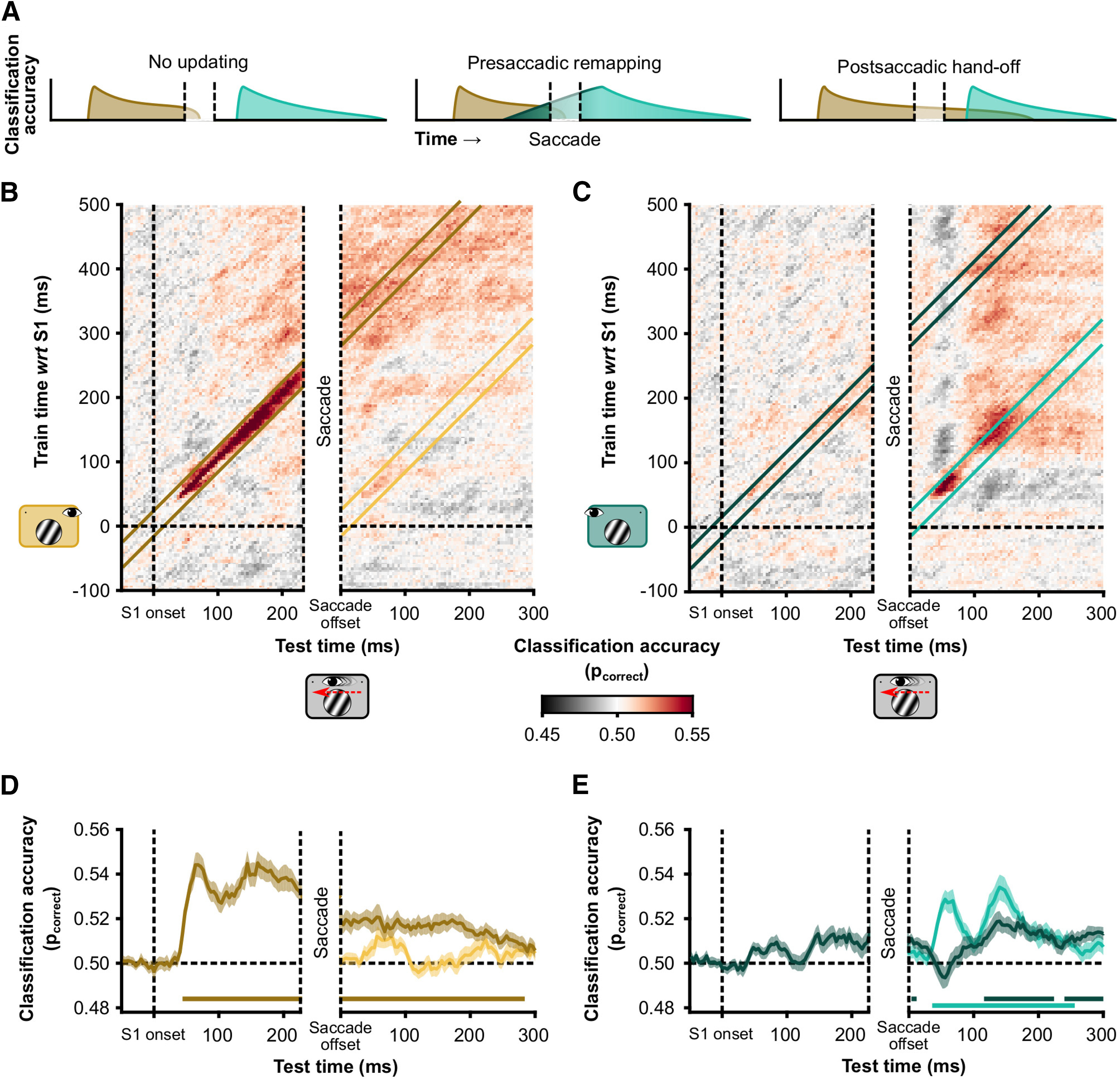
Temporal generalization of cross-decoding. ***A***, Hypothetical decoding of spatial frequency across saccades. Brown represents classification accuracy of a classifier trained on data from the Fix-LVF condition (i.e., the presaccadic classifier). Cyan represents classification accuracy of a classifier trained on data from the Fix-RVF condition (i.e., the postsaccadic classifier). We consider three hypotheses. First, spatial frequency information is not updated and is available only in retinotopic conditions (no updating). Second, the postsaccadic classifier can classify spatial frequency before saccade onset, and therefore before the stimulus location in the train data is retinotopically matched to the test data (presaccadic remapping). Third, the presaccadic classifier can classify spatial frequency well into the postsaccadic window (soft handoff). ***B***, Classifiers were trained using the Fix-LVF data, corresponding to the presaccadic visual field in the Sac-LVF condition. The Sac-LVF data were used to assess classifier accuracy. The Sac-LVF data were aligned to S1 onset (left side of the generalization matrix) and to saccade offset (right side of the generalization matrix). The width of the presaccadic window is matched to the overall median saccade latency = 226 ms. The width of the saccade window corresponds to the overall median saccade duration = 62 ms. Note that on some trials in the left half of the temporal generalization matrix the saccade had already been executed after 150 ms. Also note that limiting the presaccadic window further to 150 ms after stimulus onset would not change the interpretation. ***C***, Like ***B***, but using as training data the Fixation, right VF condition for the classifier. ***D***, For each participant, we calculated the average classification performance in two diagonal bands (illustrated in ***B***). One diagonal reflects the similarity between S1 evoked responses in the Fix-LVF condition (brown) and the S1 evoked response in the Sac-LVF condition. The other diagonal (yellow) represents the similarity between the S1 evoked response in the Fix-LVF condition and the saccade offset evoked response in the Sac-LVF condition. Lines represent the group average (*N* = 28); the shaded area represents 1 SEM across subjects. Statistical significance, indicated by the horizontal colored lines, against chance level was assessed with one-sample *t* tests on the log-odds of correct classification, corrected for multiple comparisons using cluster-based permutations with threshold-free cluster enhancement. ***E***, Like ***D*** but with the Fix-RVF as training data. Diagonals are illustrated in ***C***.

Having established that spatial frequency can be decoded from the evoked responses in all conditions, we investigated when the representation changes across a saccade (Fig. 3*A*). We used cross-condition temporal generalization to assess the time course of trans-saccadic SF representations. In this analysis, data from one condition are used to train a classifier and then the classifier is tested on data from a separate condition (i.e., cross-condition). Here, we used the Fix-LVF and Fix-RVF conditions as training data and the Sac-LVF condition as the test data (Fig. 3*B*,*C*). Temporal generalization refers to training a classifier for each time point in the train data and evaluating its performance on each time point in the test data. This allowed us to characterize at which time point, with respect to the saccade, the representation of spatial frequency in the Sac-LVF condition switches from being similar to the Fix-LVF condition and begins to resemble the Fix-RVF condition, without the need for a common reference point in time. However, we focused in particular on the on-diagonal decoding performance with respect to the following two time points: S1 onset and saccade offset (Fig. 3*B*,*C*). We used this approach instead of simply aligning the data to two timepoints, because aligning the data to saccade offset is less precise than aligning the data to stimulus onset.

#### Postsaccadic classification of presaccadic spatial frequency

Results of the cross-condition temporal generalization are displayed in Figure 3, *B* and *C*. This analysis resulted in two main findings. The first was that the classifier trained on data from the Fix-LVF condition could still decode the spatial frequency in the Sac-LVF condition well after saccade offset (cluster from saccade offset to 384 ms thereafter), when the stimulus had already been brought from the left into the right visual field (Fig. 3*D*, brown line). This finding shows that it would be possible, in principle, for higher visual areas to read out the spatial frequency of the stimulus during the entire interval, across the saccade, and well into the new fixation.

#### Rapid postsaccadic classification of postsaccadic spatial frequency

The second main observation was that cross-condition decoding of the Sac-LVF condition using Sac-RVF training data resulted in rapid on-diagonal decoding postsaccadically (Fig. 3*E*, cyan line, cluster from 36 to 256 ms). In other words, the evoked response by the spatial frequency after saccade offset resembled the response to the same spatial frequency after stimulus onset in Fixation trials. This finding suggests that the rapid increase in classification performance by the Fix-RVF classifier after saccade offset primarily used feedforward information that is retinotopically organized. Around 116 ms into the new fixation, spatial frequency information is sufficient to decode along either diagonal (from either training set; Fig. 3*E*). At this point, both the feedforward postsaccadic input and the presaccadic input provide similar information about spatial frequency.

#### Diagonal width specificity

**Figure 4.**
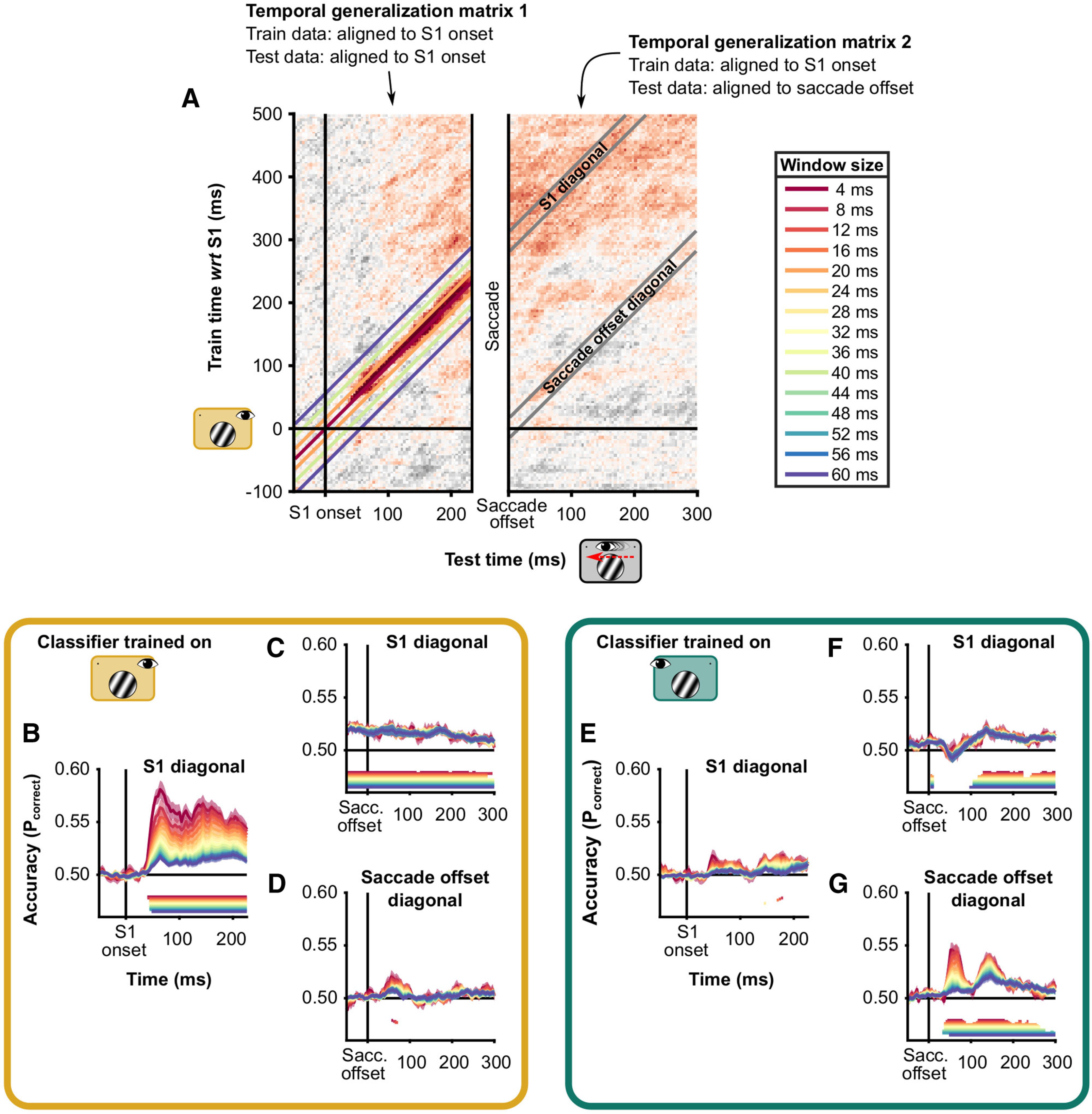
Classification on diagonals of temporal generalization. ***A***, Temporal generalization matrices as depicted in Figure 3*A*. The figure consists of two temporal generalization matrices, one where the test data (from the Saccade condition) are aligned to stimulus (S1) onset and one where the test data are aligned to saccade offset. In both cases, the classifiers were trained on data from the Fixation condition. On top of temporal generalization matrix 2, the diagonals are depicted in gray. These diagonals have a width of 20 ms; that is, they average cells of the matrix that are within 20 ms of the actual diagonal. At a temporal resolution of 250 Hz, this means five samples. On temporal generalization matrix 1, diagonals of four different widths are visualized. ***B–D***, Diagonals extracted from temporal generalization matrices where the data of the Fixation, Left VF were used to train the classifiers. ***E–G***, Like ***B–D***, but where the data of the Fixation, Right VF were used to train the classifiers. In each panel, periods with significant above-chance classification are indicated by horizontal lines, in the same color as the data.

It is important to note that these conclusions do not depend on the width of the diagonal bands, taking different bandwidths results in similar pattern of inferences (Fig. 4). The strength of retinotopically aligned classification (Fig. 4*B*,*G*) is most affected by the width of the diagonal band. Instead, the postsaccadic classification of the presaccadic stimulus is affected only slightly (Fig. 4*C*).

#### Emergent spatial invariance of spatial frequency information

**Figure 5.**
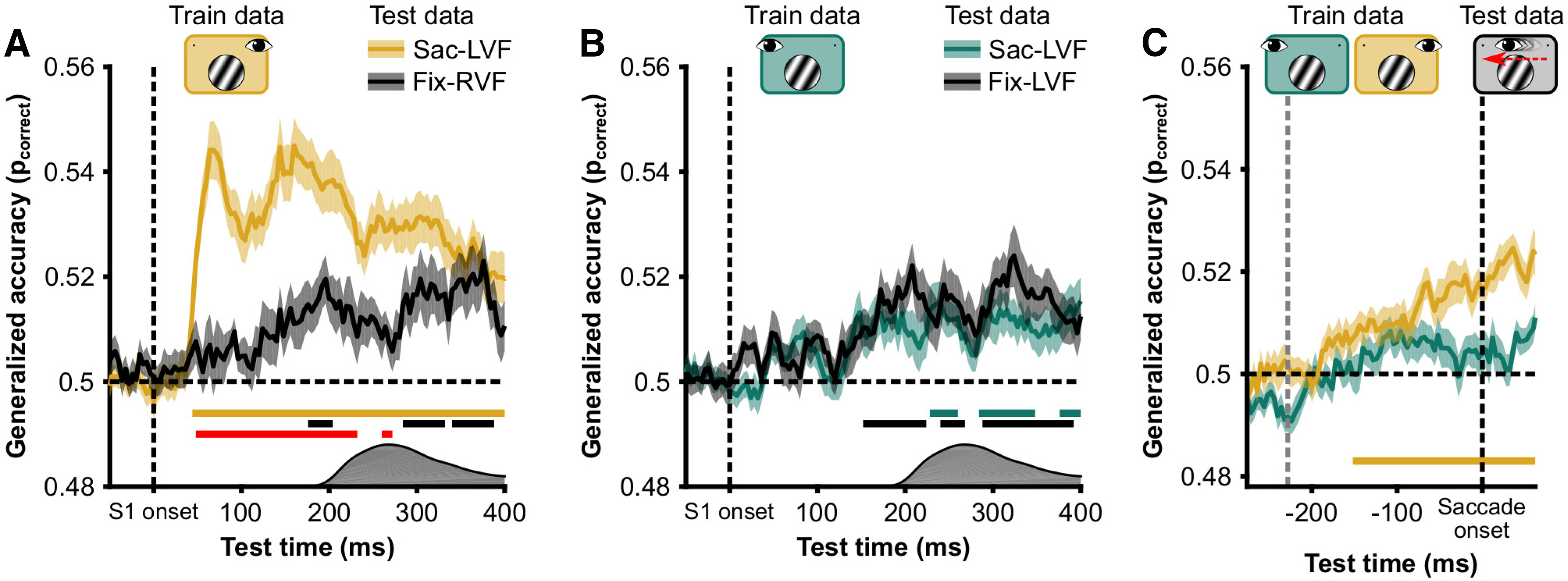
Control analyses. ***A***, Spatial invariance of spatial frequency decoding. Classifier was trained on Fix-LVF data and used to classify Fix-RVF data (green) and Sac-LVF (yellow, a replication of the brown line in Fig. 3*D*). Data presented here are diagonals from the temporal generalization matrix (as depicted in Fig. 3). Horizontal yellow and black lines indicate significant above-chance (*p*_correct_ = 0.5) classification. Red line indicates a difference in classification between Fix-RVF and Sac-LVF data. Because saccades were made in the Sac-LVF condition, but not in the Fix-RVF condition, the distribution of saccade onsets is depicted on the *x*-axis to indicate the time at which the stimulus switched visual fields in the Sac-LVF condition. ***B***, Like ***A***, but for a classifier trained on the Fix-RVF data. ***C***, Classification accuracy of a classifier trained on the Fix-LVF condition (yellow) and Fix-RVF condition (green) for classifying the Sac-LVF condition, with the Sac-LVF data aligned to saccade onset. The train data were aligned to stimulus onset. Before saccade onset, only the classifier trained on the retinotopically matched data (yellow) could classify spatial frequency in the Sac-LVF condition. Horizontal line indicates significantly above chance classification.

To assess the spatial specificity of the cross-conditions classification, we compared how accurately Fix-LVF classifier was able to decode SF from the Fix-RVF condition, compared with the Sac-LVF condition. Early after stimulus onset, classification accuracy was higher for the Sac-LVF condition than for the Fix-RVF condition (Fig. 5*A*). However, after 176 ms, both conditions could be classified above chance, with accuracies not significantly different between the two conditions after 280 ms. A classifier trained on the Fix-RVF data could classify the other two conditions from 152 ms (Fix-LVF) and 228 ms (Sac-LVF) after stimulus onset, with no significant differences between the two conditions at any time point (Fig. 5*B*). Together, this suggests that spatial frequency information becomes spatially invariant ∼200 ms after stimulus onset, allowing for the decoding of spatial frequency across the saccade and well into the new fixation.

#### No presaccadic updating of spatial frequency information

We examined presaccadic updating of visual information by classifying SF from saccade-onset aligned data of the Sac-LVF condition, with classifiers trained on the Fix-RVF or Fix-LVF data. If the classifier trained on the Fix-RVF data would be able to classify spatial frequency above chance level before saccade onset, this would be evidence for presaccadic updating. We did not observe this in our data (Fig. 5*C*). However, possibly, with longer saccade latencies, the Fix-RVF classifier could have also classified the Sac-LVF data above chance level, like it could classify the Fix-LVF data 152 ms after stimulus onset.

#### No classifier bias after saccade offset

We assessed a bias for high or low spatial frequencies by examining the predictions that the Fix-LVF and Fix-RVF classifiers made for the Sac-no VF data, with the Sac-no VF data aligned to saccade offset. At no time point after saccade offset was there a significant bias in either classifier.

## Discussion

One fundamental mystery in neuroscience is how the world appears perceptually stable regardless of the dramatic changes in retinal input that follow saccadic eye movements. Classic studies argued that most low-level information, such as SF, is suppressed and discarded with each saccade (Irwin et al., 1983; Melcher, 2005). Yet several previous studies showed feature-dependent modulations of neural responses in spatiotopic coordinates (Subramanian and Colby, 2014; Dunkley et al., 2016; Fairhall et al., 2017; Zimmermann et al., 2017), although these modulations seem dependent on the behavioral relevance of the stimulus (Lescroart et al., 2016; Mirpour and Bisley, 2016; Yao et al., 2016). Moreover, the time point at which feature-dependent modulations occur was often unclear because of temporal resolution (i.e., the BOLD response; Dunkley et al., 2016; Fairhall et al., 2017; Zimmermann et al., 2017). Here, we decoded SF across saccades from MEG data. Classification performance showed that information about SF from the presaccadic stimulus was present before and well after the saccade. The decoding results suggest that SF representations (1) develop quickly after both stimulus onset and saccade offset in retinotopic coordinates, (2) become spatially invariant after ∼150 ms and (3) can still be read out after saccade offset.

The combination of a rapid feedforward response and the ongoing presaccadic representation of the stimulus, which becomes less retinotopically specific after the first 150 ms, might play an important role in rich and continuous perception across the saccade. We found that, even under stable fixation, SF information became less retinotopically specific over time, from ∼150 ms after stimulus onset, leading to “emerging nonretinotopy” (Melcher and Morrone, 2015). This suggest that, in addition to “retinotopically lingering attention” (Golomb et al., 2010), emerging nonretinotopy of feature information could allow presaccadic and postsaccadic information to be combined and support trans-saccadic perception without requiring predictive remapping in early visual processing areas (Irwin, 1992; He et al., 2019), since higher brain areas could read out the SF of the stimulus during the entire interval. Combined with the availability of gaze position information in early visual areas (Morris and Krekelberg, 2019), low-level visual information could, in theory, be read out in head-centered coordinates continuously across a saccade (Andersen et al., 1985; Pouget and Sejnowski, 1997).

Interestingly, classification performance did not show saccadic suppression, which has been shown to reduce visual sensitivity ≥50–100 ms before a saccade and continue for 50–100 ms after the saccade (Binda and Morrone, 2018). There may have been a brief period during the actual saccadic eye movement during which decoding suffered that was not captured by our analysis. However, we should note that saccadic suppression does not refer to a shutdown of all cortical processing during a saccade, but rather to a momentary decrease in sensitivity to newly onset visual stimulation (Bremmer et al., 2009). Because we showed participants a single, stable visual feature, decoding could have been based on downstream processing rather than the suppressed early stage.

In line with the dual-spotlight theory (Golomb, 2019), a useful metaphor for the problem of visual stability is that of a mobile phone moving through a city. A new cell tower begins to provide coverage while the previous cell tower is still responding, allowing for a “soft handoff” over time and no drop in coverage. Critically, this does not require low-level information to be transferred directly between the two towers, which remains the most problematic aspect of remapping theories, but instead involves a change in information transfer between the mobile phone and the two towers. The rapid encoding and temporal overlap after saccade offset are reminiscent of such a “soft handoff” in information transfer, as was previously reported for object tracking during stable fixation (Khayat et al., 2004, 2006; Drew et al., 2014) and for trans-saccadic attentional cueing effects (Golomb et al., 2014; Marino and Mazer, 2018).

The dual-spotlight (Golomb, 2019) and attentional pointers theories (Cavanagh et al., 2010) describe which parts of the visual field are sampled preferentially, at specific times before and after a saccade. Specifically, these theories state that if attention is deployed at a location before a saccade, it will linger in the same retinotopic location after the saccade. Simultaneously, starting before saccade onset, attention will also be deployed onto the location that will be retinotopically relevant after saccade offset. After saccade offset, attentional benefits (e.g., higher accuracy, faster reaction times) are observed at two locations. Many studies have focused on the remapping of spatial attention by measuring behavioral or neural responses to briefly flashed stimuli. Because the current study contained a single salient object and did not manipulate or measure spatial attention, it remains an open question where and in which reference frame attention was allocated. As such, the current results provide a potential extension to these theories without specifically testing whether they apply in the case of a single salient object in a display.

A handoff of low-level visual information would be possible if stimulus-specific information becomes quickly available. Our estimate of 40 ms is similar to a previous study that decoded SF with MEG (Ramkumar et al., 2013). The low latency of SF-specific information in the MEG data is consistent with the classifier using signals from early visual areas. Neurophysiology studies with monkey subjects showed latencies in this order (30–50 ms) to spatial frequencies in the superior colliculus (Mazer et al., 2002; Chen et al., 2018a). This rapid postsaccadic response would allow for the readout of visual information significantly earlier than expected based on previous trans-saccadic studies measuring high-level visual information about object identity or category, which showed time scales well over 100 ms (Edwards et al., 2018; Huber-Huber et al., 2019). High-level visual information, such as facial identity, is represented by neurons with large receptive fields and requires more time to process than SF. It would be useful to predict such information in advance to update/integrate across saccades, whereas SF, involving rapid processing and smaller receptive fields, would benefit less from prediction.

Maintenance of feature information into the new fixation, while also processing new feedforward input requires some sort of multiplexing. This is not only a problem for trans-saccadic perception but also for rapid visual events presented during fixation. Quickly succeeding stimuli can be processed by and decoded from the visual system even when the succession rate surpasses the processing time of each single stimulus (Grootswagers et al., 2019; King and Wyart, 2019). How such multiplexing is implemented in the brain is a topic for further study. With respect to multiplexing around the time of saccades, there are two “ingredients” that could provide a valuable contribution: spatiotemporal modulations of receptive fields and multiplexing of receptive field profiles in different frequency bands.

First, (population) receptive field locations demonstrate a variety of spatiotemporal modulations around the time of saccades. Some visual cells respond to the future receptive field—as measured with flashed stimuli—even before the saccade onset (i.e., predictive remapping; Duhamel et al., 1992; Nakamura and Colby, 2002), but also many cells maintain the response to the presaccadic receptive field until after the saccade ended (Mirpour and Bisley, 2016; Neupane et al., 2016) which has been suggested to support postsaccadic updating (Ong et al., 2009). These different dynamics of receptive field profiles are abstracted in the dual-spotlight theory of attentional updating: with two coexisting receptive field locations, an observer is able to rapidly detect changes in visual input at the same spatiotopic location (Golomb, 2019).

Second, a study on the nonhuman primate frontal eye fields showed that information about the spatial location of a flashed target contained in the high-gamma band was compressed toward the saccade target, while alpha-band activity represented the presaccadic spatial location well into the new fixation (Chen et al., 2018b). This pattern is suggestive of multiplexing at the level of local field potentials.

Altogether, the pattern of results found here suggests that the apparent richness of perception across saccades may be supported by the continuous availability of low-level SF information that supports gist and object perception. One restriction of the current study is that we do not know the precise nature of the information content used for SF classification. Some parts could be driven by cognitive/attentional factors (behavioral performance was better for low-SF than high-SF stimuli). Future work is needed to test how the current findings generalize to other situations (e.g., different visual features, multiple stimuli, and unpredictable saccade directions).
